# Scraps

**Published:** 1888-03

**Authors:** 


### SCRAPS

PICKED UP BY THE ASSISTANT-EDITOR.

---

**Aid to Diagnosis.**—Patient, concluding recital of her case—uterine. "And, doctor, I've seen nothing for twelve months." Young physician: "One moment, madam, until I bring my ophthalmoscope."—*Communicated.*

**Unqualified Assistants.**—The General Medical Council have passed the following resolution: "That the Council record on its minutes, for the information of those whom it may concern, that charges of gross misconduct in the employment of unqualified assistants, and charges of dishonest collusion with unqualified practitioners in respect of the signing of medical certificates required for the purpose of any law or lawful contract, are, if brought before the Council, regarded by the Council as charges of infamous conduct under the Medical Act."

[A Memorandum has been issued this month referring in detail to this resolution and its scope.]

**Most Important.**—It is reported of an intelligent man who settled in a strange town, and who wished to secure a medical adviser for his family, that he went to the postmaster to find out which of the local physicians took the most medical journals. His reasoning was that the man who takes the most journals of his profession, is well read and up with the times, and consequently the one whom any sensible man would prefer to employ. Unquestionably this reasoning is sound, but does not go quite far enough. He should have discovered which of the doctors pays his subscriptions to the journals most promptly. It is the man who promptly at the beginning of the volume transmits his subscription, that is the man to tie to. Such men are the pride of the profession, and are always safe counsellors.—*The Medical Age.*

[The point of the first part of the foregoing paragraph should be put before non-subscribers to this *Journal.* Attention to the latter part would save a vast amount of unnecessary work. Last month I had to write to 127 subscribers, asking for arrears for periods varying from one to five years.]

**A Good Appetite.**—Somewhere in the neighbourhood of Aylsham lived a certain Jerry Eke, whose appetite was said to be superhuman, and whose prowess at harvest suppers was the boast and wonder and envy of the villagers round. It came to pass that at a farmers' market dinner the talk turned upon Mr. Eke's performances, when some one present protested that what had been narrated was impossible. "Impossible!" said another. "I'll bet you five pounds Jerry Eke will eat a calf at a sitting." The wager was taken, and the preliminaries were arranged. The calf—let us hope only a baby calf—was killed; the bones were cut out, the flesh was chopped into minute particles, and apportioned into seventeen enormous *pasties,* whose outer crust was a thin film of batter made lovely and tempting to every sense, but carefully kept from any ingredients that could cloy the palate. Jerry was called in, he having agreed to the wager with evident delight, and was told he might fall to. He did so, and steadily gorged. He had made no difficulty of the first nine pasties; but when a tenth was brought in he seemed to flag. To the horror of his backers, he sighed and looked perplexed. It was but for a moment; he desired only to expostulate. "I say, Mas'r, I ain't got nothing to say agin them *poys*—I loik 'em amazin'; but I'm a-thinkin' et's abaywt time as I should *begin upon that ther calf!*"—Rev. Dr. Jessopp in *The Nineteenth Century.*

**The First Day's Diary of a Baby.**—2 a.m. Born a few minutes ago. Yelled.

2.15. Am washed. The fool doctor told 'em I was a boy, just as if that was something new. Was whacked over the lap of a dizzy old Christmas card of a nurse, who proceeded to tog me out in some bandages and a quarter of a mile of skirts. Kicked.

3.0. Have slept somewhat. The gorgeous old valentine made for me, when I stirred, turned me into nineteen different positions. Must be training me for a contortionist. Yelled.

4.0. Have worked the sound wave for a straight hour. The old man isn't looking as happy as he did. I am a high soprano, I know, for I just heard someone in the fourth story swearing. Old man has remarked that I'll depreciate property for four blocks.

4.10. Everybody is sitting around. The old man has just gotten even with the doctor by giving him one of his cigars. The doctor will have to charge himself up with a prescription pretty soon.

4.11. Told you so! The doctor has just asked the old man if he had ever matched one of his cigars against a glue factory. Yelled in sympathy.

4.15. The amiable old Easter memorial is working a bottle. She saw me watching her, and said I was a tootsy-wootsy. I wish I were a shoesy-bootsy. I'd fix her for getting a corner on the family supplies and stowing them away in her stomach.

4.18 to 5.18. Yelled.

5.20. The antique circus-poster fed me on warm water and whisky. She said I had the colic. Will work the colic racket again.

6.0. Wazzer mazzer wiz ev'body? Giddy old chromo wiz two heads, whackin' me on the back. Had colic twice.

9.0. Woke up with the headache. The old man ought to keep better goods. Guess I'll yell.

9.15. Am washed. Feel a little rocky. Ten minutes for refreshments, then I intend to do the colic gag over again for a cocktail.

10.0. Old man is writing telegrams about me. He looks a little like a last year's bird's-nest himself. Yelled.

12.0. Have been asleep. Woke up suddenly and saw the venerable nightmare they've hired to groom me, working her jaws over enough lunch to feed a shift of section hands. The old man oughtn't to allow it. What'll I do when he kicks out if this waste continues? The thought made me so mad that I yelled.

3.0 p.m. Have dozed. Everybody is doing well but the people in the block, who are tired out for want of sleep. Old man has confidence in me. He has just said that he'd back my lungs against any steam whistle in town, best two toots out of three. It makes one proud to have the approval of his parents.

5.0. I was put on a pillow in a chair a few minutes ago, and a fool girl came in and sat down on me. Yelled.

5.20. Colic. Fortunate results; sleep.

8.10. Going to sleep for the night. The giddy old obelisk is in the chair snoring. Room sounds like a round house. Mighty dull sort of a day—Good-night.—*The Rambler (Cincinnati Lancet and Clinic).*

[The experience recorded above is akin to that of Dugald Stewart who "was once asked what was the earliest thing he could remember. He said it was being left alone by his nurse in the cradle, and resolving to tell of her as soon as he could speak."—SIR F. POLLOCK'S *Personal Remembrances*, vol. ii., p. 188. Neither record, however, equals that of the American who remembered that, in his early embryo-life, he had a fear that, perhaps, he might be born a girl.]

